# Ninth International Medical Congress, Washington, D. C., September, 1887

**Published:** 1888-05

**Authors:** 


### NINTH INTERNATIONAL MEDICAL CONGRESS, WASHINGTON, D. C. SEPTEMBER, 1887.

### SECTION XVIII, DENTAL AND ORAL SURGERY.

Reported for the Independent Practitioner, by "Mrs. M. W. J."

Concluded From Page 208.

#### CLINICS.

The Franklin School Building, corner of 13th and K Sts., was devoted to clinics, the exhibition of appliances, and the exhibits of the several dental manufacturing companies.

The basement was fitted up with everything necessary for clinics in prosthetic dentistry. Here Dr. L. P. Haskell, of Chicago, and Dr. John Allen, of New York, gave clinics in continuous gum work ; Dr. J. Hall Lewis, of Washington, D. C., in gold plate work; Dr. W. W. Evans, of Washington, D. C., in zylonite and celluloid; Dr. C. C. Carroll, of Meadville, Pa., in cast aluminum plate and bridge work; and Dr. J. J. R. Patrick, of Belleville, Ill., his method of making gold crowns.

On the first floor all the desks and benches had been removed from the different school rooms, and twenty-four operating chairs, each one placed inside of a low railing protecting operator and patient from the crowd, amphitheatre platforms having been erected for the convenience of spectators.

On the floor below were found the displays of the S. S. White Co., the Welch Dental Co., R. S. Williams, The American Dental Mfg. Co., The Wilmington Co., and the dental specialties of Seabury & Johnson. The water power in the building was not sufficient to run the water motor of Dr. Campbell, of Edinburg, Scotland, which was exhibited in the court of the Arlington Hotel.

Among the appliances exhibited were the Interdental Splints of Dr. Wm. Carr, of New York ; a Bandage for the Correction of Irregularities, by Dr. V. E. Turner, of Raleigh, N. C.; a Cleft Palate Obturator, by Dr. W. B. McLeod, of Edinburg, Scotland ; Dr. Genese's Anatomical Articulator, and his Syphon and Speculum.

Dr. V. H. Jackson, of New York, also had a large collection of appliances for regulating, with casts, models, etc.

Dr. J. J. R. Patrick, of Belleville, Ill., had a new catch for attaching a piece of tape to the rubber dam for each patient, in place of the elastic ribbon in use; also a shield for protecting the gums while trimming stumps for artificial crowns.

Drs. H. A. Parr, of New York; E. B. Call, of Peoria, Ill.; H. C. Merriam, of Salem, Mass.; T. S. Waters, of Baltimore; J. J. R. Patrick, of Belleville, Ill.; E. Parmly Brown, of Flushing, N. Y.; J. R. Knapp, of New Orleans, La.; S. S. Stowell, of Pittsfield, Mass.; E. T. Starr, of Philadelphia; J. S. Thompson, of Atlanta, Ga.; and S. Ludwig, of Chicago, gave clinics in crowns and bridge work. Dr. J. A. Daly was present with his all gold lining for vulcanite plates.

Dr. Younger, of San Francisco, Cal., Dr. Louis Ottofy, of Chicago, and Dr. G. L. Curtis, of Syracuse, N. Y., implanted teeth by the "Younger" method.

Dr. Geo. H. McCausey, of Janesville, Wis., and Dr. B. H. Smith, of Baltimore, used an aqueous solution ($\frac{1}{1000}$) of hydronaphthol, as a germicide and disinfectant in treating putrescent pulps.

Dr. J. P. Geran, of Brooklyn, N. Y.; Dr. E. S. Niles, of Boston, Mass., and Dr. C. A. Timme, of Hoboken, N. J., demonstrated the Herbst method, with the Wolrab gold. Dr. Geran also used cylinders and soft foil with Bonwill's mechanical mallet, in combination with the Herbst method, using 66 grs. of gold in building up a tooth, the cavity involving the whole of the masticating surface of a second molar.

Dr. E. L. Swartwout, of Utica, N. Y., used the Wolrab gold for lining cavities, packing with Watt's crystal gold.

Dr. B. H. Smith, of Baltimore, crowned four central incisors, cutting off the teeth, taking out the live pulps with broaches while benumbed or paralyzed by the shock, filling the roots and placing the crowns on the stumps at one operation.

Among the contour operators were Drs. R. F. Ludwig, who used Whitefield's electric engine, and Dr. G. S. Salomon, of Chicago, the latter using Kearsing's foil, with the Detroit Motor Co.'s battery motor and the Bonwill-Webb mallet; Dr. Wm. Crenshaw, of Atlanta, Ga., who made a series of eight or ten consecutive contour operations in the left upper jaw, restoring teeth which had been severely cut away with files, etc.; R. H. Woodhouse, of London, Eng.; Dr. Sprenkle, of Culpepper, Va.; Dr. M. C. Marshall, of Little Rock, Ark.; Dr. H. F. Harvey, of Cleveland, Ohio, using gold and platinum ; Dr. G. H. Chewning, of Fredericksburg, Va., using Quarter Century foil ; Dr. T. S. Waters, of Baltimore, using No. 30 Cohesive foil with the electric mallet ; Dr. Wm. Barker, of Providence, R. I.; Dr. C. S. Carr, of Jackson, Mich., in filling a very large cavity used tin and gold, Robinson's felt foil folded together, and an articulating face of platinum gold ; Dr. S. B. Price, of New York, using smooth point pluggers with soft foil ; Dr. Hofheinz, of Rochester, N. Y., made a filling with cylinders; Dr. E. C. Moore, of Detroit, Mich., used Williams' soft foil cylinders; Dr. Geo. W. Whitefield, of Evansville, Ill., operated with Steurer's plastic gold; Dr. L. L. Davis, of Eaton Rapids, Mich., used the electro-magnetic mallet; Dr. H. A. Parr, of New York, demonstrated the value of his Universal Separator; Dr. R. B. Adair, of Gainesville, Ga., treated a number of cases of pyorrhœa alveolaris by his new method; Dr. Wm. H. Richards, of Knoxville, Tenn., treated cases of antral abscess, and Dr. Geo. W. Whitefield illustrated his method of bleaching discolored teeth by passing a current of electricity through common salt, packed in the cavity, liberating chlorine, and thus setting free oxygen, the bleaching agent, in the tubuli. Dr. Shumway gave several clinics with his ivory-pointed pluggers.

Interest in the clinics was unabated to the very last, several operations being performed on Saturday, the day after adjournment of the Congress. Indeed, the Franklin School building seemed to be the central point of interest of the section. As early as eight o'clock A. M. it was usually thronged, and the operators were at work, and from that time during the whole day, except the hours devoted to the sessions, it was difficult to make one's way through the crowded rooms. When the time came for opening the sessions, the officers and committees had hard work to stop the progress of operations, and the crowd lingered until after the last operator closed his case and took off his office coat. Foreign members of the section were especially anxious to witness the clinics of which they had heard so much. American dentistry was known to be intensely practical, and American operators to be the best in the world, full of original conceits and fruitful in ingenious devices. Many foreign dentists had seen fine fillings that had been inserted in America, and were anxious to learn the exact *modus operandi.*

American dentists are always interested in clinics. They belong to a practical people, who have been chiefly engaged during the comparatively brief period of their national history in the solution of urgent industrial questions, and this has given a turn to national enquiry and experimentation. Dentistry in America was early thrown on its own resources, and obtaining no recognition from its scientific mother, Medicine, it at once became engaged in a struggle for professional existence, and that, too, has tended to make its character real and practical. It is little wonder, then, that from this national and international spirit of enquiry, and the known character for originality and inventive genius of American dentists, the practical part of the dental section of the Ninth International Medical Congress should have been of great interest. Nor did any one express any disappointment in the exhibitions. American operators are accustomed to work before an audience, and habituated to the necessary explanations, and the clinics maintained their attractions to the last.

Of all the demonstrations of operative dentistry, perhaps nothing attracted more universal attention than implantation. It was exemplified by Drs. Younger, Ottofy and Curtis. Dr. Younger, as the one who introduced it to the profession of to-day, and who has enjoyed greater experience in it, was, of course, the first favorite. He is a dashing, daring, brilliant, rapid operator, of striking appearance and confidence-begetting mien. Perhaps his operations were not more skillfully conducted than those of the others, but the interest centered in him, and a number of members of the Section of General Surgery attended his clinics, and gave the operation close attention.

The exhibits of the different dental depots also attracted great attention, especially from the foreigners present. There were so many ingenious helps that were new to them, and so much of American methods of practice could be studied from an examination of the implements with which they performed their wonders, that the rooms of the principal exhibitors were constantly crowded. The small army of the employees of the S. S. White Co. especially, were kept constantly busy in answering questions and explaining appliances.

The gold exhibit of R. S. Williams attracted universal attention, and his display was much admired.

The display of Seabury & Johnson did not receive the attention which its just merits demanded, for it was not in a conspicuous location. The exhibit of the appliances of Dr. Parsons, of Wamego, Kansas, was in the same room, and that too did not get its just deserts.

In addition to the American exhibitors, the great house of C. Ash & Sons, of London, who have a branch house in New York (as indeed they have in most of the important dental cities of the world), showed a large stock of their manufactures, and it was very instructive to compare English with American instruments. Sometimes this was greatly to the advantage of America—and sometimes not—but to the practicing dentist the benefit was the same, whichever might claim superiority.

The rooms of the other exhibitors named in this report were constantly thronged, and the displays much admired. There was not one which did not have some special features of interest that attracted the attention, not alone of the dentist, but of the members of other sections, and of the non-professional visitor as well.

---

### CENTRAL DENTAL ASSOCIATION OF NORTHERN NEW JERSEY.

### REGULAR MEETING FOR OCTOBER, 1887.

REPORTED FOR THE INDEPENDENT PRACTITIONER.

